# A Review of the Aetiopathogenesis and Clinical and Histopathological Features of Oral Mucosal Melanoma

**DOI:** 10.1155/2017/9189812

**Published:** 2017-05-30

**Authors:** Liviu Feller, Razia A. G. Khammissa, Johan Lemmer

**Affiliations:** Department of Periodontology and Oral Medicine, Sefako Makgatho Health Sciences University, Medunsa, Pretoria 0204, South Africa

## Abstract

Oral mucosal melanoma is an uncommon, usually heavily melanin-pigmented, but occasionally amelanotic aggressive tumour with a poor prognosis. Despite radical surgery, radiotherapy, or chemotherapy, local recurrence and distant metastasis are frequent. Microscopical examination is essential for diagnosis, and routine histological staining must be supplemented by immunohistochemical studies. The aetiology is unknown, the pathogenesis is poorly understood, and the 5-year survival rate rarely exceeds 30%. In most cases, oral mucosal melanoma arises from epithelial melanocytes in the basal layer of the epithelium and less frequently from immature melanocytes arrested in the lamina propria. In both cases the melanocytes undergo malignant transformation, invade deeper tissues, and metastasize to regional lymph nodes and to distant sites. Very rarely metastasis from skin melanoma may give rise to oral mucosal melanoma that may be mistaken for primary oral mucosal melanoma. The pathogenesis of oral mucosal melanoma is complex involving multiple interactions between cytogenetic factors including dysregulation of the cKit signalling pathways, cell cycle, apoptosis, and cell-to-cell interactions on the one hand and melanin itself, melanin intermediates, and local microenvironmental agents regulating melanogenesis on the other hand. The detailed mechanisms that initiate the malignant transformation of oral melanocytes and thereafter sustain and promote the process of melanomagenesis are unknown.

## 1. Introduction

Oral mucosal melanoma is a rare and ultimately fatal cancer originating from malignant transformation and clonal expansion of neural crest-derived melanocytes residing either in the basal cell layer of the oral epithelium or in the lamina propria of the oral mucosa. The five-year survival rate seldom exceeds 32% [1–3].

Oral mucosal melanoma constitutes about 25% of all mucosal melanomas of the head and neck [4, 5], about 2% of all mucocutaneous melanomas [6], and about 0.5% of all oral malignancies [1, 7]. It is more prevalent in Japanese, African, and American indigenous populations than in Whites [3, 7, 8].

According to various publications, between 2% and 40% of all oral mucosal melanomas are amelanotic. Although the clinical and epidemiological features of melanotic and amelanotic melanoma are similar, the prognosis of subjects with amelanotic mucosal melanoma seems to be poorer than those with melanotic melanomas, probably because, owing to the absence of pigment, the diagnosis of the former is more difficult, thus delaying the commencement of treatment [9].

The aetiology of oral mucosal melanoma is unknown: there is no evidence that repetitive trauma, chronic inflammation, human papilloma virus (HPV) infection, or tobacco smoking plays any role in its pathogenesis. The only known risk factor is the preexistence of a mucosal field of melanin hyperpigmentation [7].

Although the prognosis for oral mucosal melanoma is never good, indicators of a dire prognosis for established melanoma include a tumour thickness of greater than 6 mm, metastatic involvement of cervical lymph nodes, presence of distant metastases, and the presence of exophytic ulcerated lesions and a high tumour cell mitotic rate [1, 7].

As oral mucosal melanoma is a complex and relatively rare malignancy, and as most data are derived mainly from retrospective studies and from series of reports of small numbers of cases, each using different clinical staging systems, the evidence-based information about risk and aetiological factors, genetic factors associated with the initiation and progression of the disease, and prognostic factors and outcomes of different treatment modalities, is scarce. Hopefully this paper may shed light on various aspects of oral mucosal melanoma.

## 2. Oral Mucosal Melanoma Precursor Cells

Most primary oral mucosal melanomas arise from melanocytes in the basal cell layer of the oral epithelium but some from immature melanocytes residing in the lamina propria of the oral mucosa.

Oral melanocytes are derived from neural crest stem/progenitor cells, most of which during embryogenesis migrate to their final destination in the basal cell layer of the oral epithelium. The migration of precursor melanocytes and their differentiation into mature, melanin-producing, or amelanotic melanocytes are mediated by a number of biological agents and intracellular signalling pathways including stem cell factor (SCF) and its tyrosine kinase receptor cKit signalling pathway, endothelins 1 and 3, hepatocyte growth factor, and basic fibroblast growth factor [10]. However, for reasons unknown, in their pathway of migration towards the oral epithelium, some immature melanocytes become arrested in the lamina propria [11].

The functions of oral melanocytes are not well understood; they produce melanin which contributes to the colour of the oral mucosa, provides protection from stressors such as reactive oxygen species and free radicals, and has the capacity to sequester metal ions and bind organic molecules [10]. Apart from their role in melanin biosynthesis, melanocytes can act as immunocytes producing cytokines, thus having the capacity to modulate immunoinflammatory responses, and as neuroendocrine cells secreting melanocortins, opioids, catecholamines, and acetylcholine into the local microenvironment. These functions of oral melanocytes all contribute to the homeostasis of the oral tissues [10, 12].

Signals from neighbouring keratinocytes, biological agents released from intramucosal nerve sensory endings, mesenchymal-epithelial interactions, and autocrine signals all together play a role in regulating the functional activity of oral melanocytes [11].

Although the population of oral melanocytes appears to remain more or less constant throughout life, the mechanisms for their replacement in the basal cell layer of the epithelium after loss to physiological processes of apoptosis, or to mechanical, thermal, or chemical injury, are not well understood. This replacement may be either the result of division of “mature” melanocytes in the basal cell layer of the oral epithelium which still retain the capacity to replicate or the result of migration of melanocyte stem/progenitor cells from local microenvironmental niche reservoirs. However, niche reservoirs of tissue-specific stem/progenitor cells containing the genetic information and the regenerative capacity of oral melanocytes have not as yet been identified [13].

The origin of the melanoma precursor cell is open to debate, but it is most probably a tissue-specific stem/progenitor melanocyte that over time has acquired cytogenetic and epigenetic alterations and has ultimately undergone malignant transformation. Melanoma stem/progenitor cells like normal melanocyte progenitor cells maintain their undifferentiated phenotypes and their capacity for self-renewal. Alternatively, it is also possible that melanoma precursor cells, either in the basal cell layer of the epithelium or in the lamina propria of the oral mucosa, are derived from melanocytes having undergone a process of dedifferentiation subsequently to cytogenetic alterations, acquiring a melanoma stem/progenitor cell phenotype. These melanoma stem/progenitor cells are the driving force behind the ongoing growth of the primary tumour and behind the establishment of distant metastases. Their replication provides new melanoma transient-amplifying cells that have a high proliferative rate and promote tumour growth [14, 15].

## 3. The Pathogenesis of Oral Mucosal Melanoma

**Figure 1:**
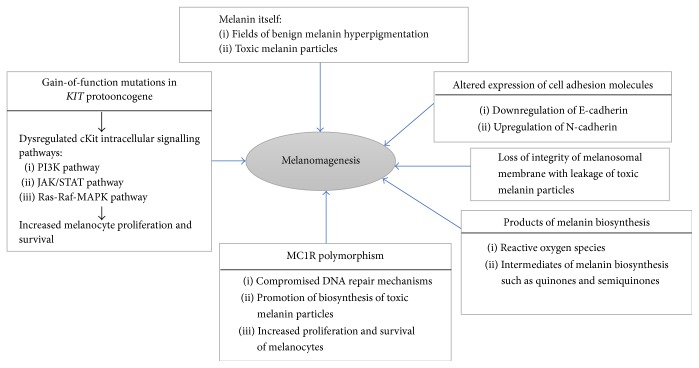
Mechanisms of melanomagenesis of oral mucosal melanoma.

Oral mucosal melanomagenesis is not associated with any known carcinogenic agents [16]. Only some of the intracellular molecular signalling pathways that drive the complex process of mucosal melanomagenesis have been identified. However, it is probable that dysregulation of cell cycle progression, apoptosis, cell-to-cell interactions, cKit signalling pathways, melanocortin receptor 1 (MC1R) polymorphism, melanin itself, and products of melanin biosynthesis are all risk factors (Figure 1). Biological agents in the microenvironment which under physiological conditions regulate the proliferation, differentiation, and function of local melanocytes also appear to play a role in the process of oral melanomagenesis [14].

The activation of the receptor tyrosine kinase (RTK) cKit by its ligand the stem cell factor (SCF) induces intracellular signalling pathways which are critical to melanocyte development and migration during embryogenesis and, later, postdevelopmentally, to the regulation of melanocyte proliferation, differentiation, and survival as well as for mediation of melanin biosynthesis [16–18]. cKit, is a product of* KIT* protooncogenes, is a transmembrane glycoprotein, and is expressed by a variety of cells including melanocyte precursors, haematopoietic progenitor cells, and mast cells [19, 20]. The RTK cKit downstream intracellular signalling pathways include the PI3K (phosphoinositide 3′-kinase) pathway, the Janus Kinases (JAKs)/Signal Transduction and Activator of Transcription (STAT) pathway, and the Ras-Raf-MAPK (p38, ERK 1/2, JNK) pathways [21].

Up to 40% of mucosal melanoma cells either overexpress the cKit protein or express gain-of-function mutations in* KIT* protooncogenes, or both. These molecular alterations may subsequently activate the Ras-Raf-MAPK transduction pathway resulting in increased proliferation and survival of melanoma cells [19, 22, 23] (Figure 1). Oral mucosal melanoma cells however show only infrequent mutations in either the NRAS or the BRAF molecular pathways that are common in melanoma of sun-exposed skin [14, 23].

In oral mucosal melanoma the dysregulated increased activation of cKit signalling pathways is not owing to upregulation of autocrine secretion of the cKit ligand SCF but rather to ligand-independent constitutive activation of cKit receptor tyrosine kinase secondary to gain-of-function mutations of the* KIT* protooncogene [17]. This results in receptor autophosphorylation with the activation of the downstream Ras-Raf-MAPK signalling pathways [20].

Many mucosal melanoma cells overexpress the cKit protein but only in some of these cells does activating mutation of the* KIT* oncogene occur; and some oral mucosal melanomas that express* KIT* mutations do not in fact overexpress the encoded protein. This is probably because overexpression of cKit protein can also be induced by autocrine/paracrine stimulation, increased copy number of the* KIT* gene, and epigenetic factors [17, 24]. Some of the activating mutations encode nonfunctional rather than functional proteins and these nonfunctional proteins account for the overexpression of cKit receptor; and not all gain-of-function mutations result in dysregulated increased melanocyte proliferation and survival. Thus, the biopathological significance of cKit overexpression or of activating mutations of the* KIT* oncogene in oral mucosal melanomagenesis has not been fully determined [17, 18], and it is very likely that other factors must interact with the dysregulated cKit-induced signalling pathways for the cKit to exert an oncogenic effect [21, 24].

Melanocortin 1 receptor (MC1R) plays a role in melanin biosynthesis. Its activation by proopiomelanocortin (POMC) and its derivates, particularly *α*-melanocyte stimulating hormone (*α*MSH), mediates the production of both brown/black eumelanin and yellow/red pheomelanin. MC1R induced intracellular signalling pathways in melanocytes can also mediate immunoinflammatory responses in the microenvironment, regulate proliferation and survival of melanocytes, and promote DNA repair of damaged DNA caused by oxidative stresses [10].

The MC1R gene is highly polymorphic with some genetic variants associated with reduced capacity for DNA repair or for apoptosis and with the generation of oxidative stress secondary to dysregulated melanin production. Thus, melanocytes with certain MC1R variants may be at increased risk of malignant transformation [25].

Altered expression of the cadherin cell adhesion molecules has also been implicated in the pathogenesis of oral mucosal melanoma. In melanoma cells there is abnormal downregulation of expression of E-cadherin molecules and upregulation of expression of N-cadherin molecules that probably contribute to the increased proliferation, migration, and invasive potential of melanoma cells [26].

As outlined above, melanin itself provides protection against reactive oxygen species and toxic free radicals; but, on the other hand, the process of melanin biosynthesis itself, particularly that of pheomelanin, has the capacity to generate reactive oxygen species that may cause DNA damage; and intermediates of melanin biosynthesis such as quinones and semiquinones are potentially mutagenic and as such can promote cytogenetic instability. Therefore, loss of the integrity of melanosome membranes with leakage of toxic melanin particles, intermediates of melanogenesis and reactive oxygen species into the cytoplasm and nucleoplasm of melanocytes, may render these melanocytes prone to an increased risk of DNA damage and consequently to malignant transformation (Figure 1) [10].

As about 30% of all cases of oral mucosal melanoma arise within fields of benign or physiological melanin hyperpigmentation, it is reasonable to assume that the dysregulation of melanin biosynthesis by hyperactive melanocytes and the increased cytoplasmic and nucleoplastic content of melanin and byproducts of melanin synthesis may play roles in the initial transformation of epithelial melanocytes [14, 27–29].

With regard to oral mucosal melanomas which arise de novo, most of them are densely to very densely melanin-pigmented, but it is not known whether the increased production of melanin is an early biopathological event in melanomagenesis or whether it is a late biopathological event associated with acquisition of a malignant phenotype by the proliferating atypical initially transformed melanocytes. In any case, melanin itself seems to be a risk factor for melanomagenesis.

## 4. Clinical Features

Clinically, the lesions of early oral mucosal melanoma are usually painless, irregularly shaped, brown to black macules or papules which may progressively enlarge, growing into nodules or exophytic masses and gradually becoming more deeply pigmented. Advanced lesions may be painful, ulcerated, and fragile and may bleed readily. The pigmentation is usually nonuniform with mottled shades of grey, dark blue, dark brown, or black [6, 30]. Sometimes multiple independent melanomas may arise within a limited field of restricted oral epithelium harbouring atypical melanocytes that have undergone malignant transformation [14, 30]. It appears that there is a higher risk of regional metastasis from exophytic ulcerated than from maculopapular oral mucosal melanomas [9].

Oral mucosal melanoma usually develops de novo in clinically normal-looking mucosa, but it has been reported that in up to one-third of cases it arises from within areas of benign oral melanotic hyperpigmentation of the oral mucosa [6]. It most commonly (about 80%) affects the palate and the maxillary gingiva, followed by the retromolar region and the buccal mucosa. It affects males and females in their 5th to 7th decades of life equally, though some studies have shown a slightly higher male prevalence [1, 2, 8, 9]. A list of differential diagnoses of oral mucosal melanoma is given in Table 1.

About 25% of subjects with oral mucosal melanoma already have regional lymph node metastases at the time of diagnosis, and about 10% have distant haematogenous dissemination to the lung, liver, bone, or brain [9, 14].

## 5. Histopathological Features

Oral mucosal melanoma can be classified into three microscopic patterns: in situ, invasive, and combined. The tumour usually starts with radial proliferation of atypical melanocytes in the basal cell layer of the epithelium. This is followed by a phase of vertical growth of invasive nodular aggregates of atypical melanocytes in the lamina propria. In a combined lesion, the radial in situ and the vertical invasive nodular patterns of malignant proliferation can be observed ab initio [1]. However, if a melanoma arises from an atypical immature melanocyte, or melanocytes residing in the lamina propria, it will initially proliferate forming nodular aggregates in the lamina propria/submucosa before invading more widely and metastasizing [1, 7, 14].

Melanoma cells may be polyhedral, round, fusiform, epithelioid, spindle-shaped, or pleomorphic. Their size varies and their nuclei contain one or more eosinophilic nucleoli: mitotic activity is a prominent feature. Proliferating melanoma cells form either solid, loosely cohesive, pseudoalveolar, or organoid patterns [9]. In about two-thirds of cases variable amounts of melanin can be detected either in the tumour cells, in macrophages, or as free extracellular particles. In some cases, the amount and density of the pigment are enough to obscure the morphology of the tumour cells [14]. Those melanoma cells without melanin are referred to as being amelanotic.

Oral mucosal melanoma cells express the melanocytic markers MART-1/Melan-A, HMB-45, MITF, tyrosinase, and S-100 protein to varying degrees. These markers can be detected immunohistochemically. However, as their sensitivity and specificity are not absolute, no single marker should be relied upon, but a battery of tests should be undertaken in attempting to confirm a diagnosis of a suspected oral mucosal melanoma [9].

## 6. Staging and Principles of Treatment

Once a diagnosis of oral mucosal melanoma has been made, comprehensive clinical and special investigations must be done to determine whether the oral melanoma is primary or metastatic and, if primary, the extent of local invasion and whether or not there is spread to regional lymph nodes or to distant sites [1, 9].

The most commonly used system of classification for staging of mucosal melanoma of the head and neck is the melanoma staging system of the American Joint Committee on Cancer (AJCC) (Table 2). In this TNM (tumour/lymph node/metastases) staging system, owing to the aggressive histopathological behaviour of oral mucosal melanoma, the lowest T (tumour) category is T3 which is associated with clinical stage III (Table 2). Oral mucosal melanoma in situ is regarded as being already a T3 and should thus be treated as an invasive melanoma [1, 2, 9].

It appears that the tumour clinical stages according to the AJCC correlate well with observed prognosis and provide predictive information about survival rates [1, 9, 31]. For stages III/IVa, complete surgical resection, with tumour-free margins of 1-2 cm, is the mainstay of treatment. Unfortunately, more than 50% of surgically treated subjects, even though their resection margins were apparently clear, develop distant metastases relatively soon after surgery and die. Realistically however, although surgical intervention does not substantially increase the overall survival rate, it remains the first line of treatment, and even in cases of postsurgical recurrent local disease without distant metastases further resection is the best option [1, 9].

Although melanoma cells have long been considered to be resistant to radiotherapy, new modalities of radiotherapy appear to be more effective so that postsurgery adjuvant radiotherapy improves control of local disease. Unfortunately, this does not translate into improvement of overall survival rates [2, 3, 9], probably because distant spread of primary oral melanoma cells with a metastatic genotype occurs early in the course of the disease.

Radiotherapy alone should be reserved for palliation of primary and recurrent oral mucosal melanomas that are beyond surgical treatment [9]. No evidence-based data show that any cytotoxic agents and/or biological immunomodulatory agents are effective in the treatment of oral mucosal melanoma. However, for lack of any better treatment, these agents have been used in the management of subjects with disseminated (stage IVb) or with advance recurrent disease as a palliative measure [1–3, 9].

In addition, for those oral mucosal melanoma cells with* KIT* activating mutations, treatment with cKit inhibitors which target the cKit intracellular signalling pathway may be beneficial [32]. However, as in mucosal melanoma activating mutations of the* KIT* oncogene can occur at each of the gene's several exons [20, 33], target therapy may be successful only if the targeted molecules of the molecularly altered receptor protein are expressed by tumour cells. Furthermore, the gene mutations that bring about the molecular amino-acid sequences cannot always be targeted by the drug [34]. In fact, subjects with oral mucosal melanoma with tumour cells which stained positively for cKit protein but without* KIT* gain-of-function mutations do not respond to treatment with cKit inhibitors [19].

The lack of a significant response of oral mucosal melanoma to targeted molecular therapy is probably because the pathogenesis of the disease involves concurrent interactions between several molecular pathways and that several oncogenic events are required for the development of occult disease. Therefore, targeting a single molecule of one specific dysregulated oncogenic pathway is not sufficient to bring about beneficial effects [23].

Neck dissection is necessary in any case of lymph node involvement by metastatic oral melanoma, and as about 50% of cases where there were no regional metastasis at the time of diagnosis eventually develop nodal disease, initial elective dissection of the neck is recommended in most cases [1, 9]. Although there are no evidence-based guidelines available, and despite the fact that radiotherapy is not effective in the treatment of oral mucosal melanoma, subjects with extensive nodal metastasis are not infrequently given radiotherapy in the hope of reducing distant dissemination [9].

## 7. Distant Metastasis of Oral Mucosal Melanoma and Melanoma Metastasis to the Mouth

In general, the genetic profile that imparts to certain cancer cells in a primary tumour the capacity to metastasize may be expressed early in the process of cancerization or may be acquired only later in the course of growth of the primary cancer, secondary to evolution of multiple subclones of cells that have undergone many additional episodes of cytogenetic and epigenetic alterations [36]. Regardless of whether they leave the primary tumour at an early or at a late stage of its growth, oral mucosal melanoma cells almost invariably metastasize to distant sites by haematogenous spread [36].

Primary cancer cells with a metastatic genotype which disseminate early often remain dormant in their new location for variable periods. The dormant metastatic cells will become active only if a favourable local microenvironmental niche develops [36]. The development of such a favourable metastatic niche is mediated by biological agents secreted by the primary cancer-associated stroma cells that accompany the metastatic cells, by haematopoietic progenitor cells, by endothelial progenitor cells, and by fibronectin [37].

Very rarely oral mucosal melanoma is not a primary melanoma but is metastatic from a distant site. Clinically it is impossible to distinguish between primary and metastatic oral mucosal melanoma but it has been reported that metastatic oral melanoma, if it occurs, affects the floor of the mouth and the tongue which are seldom affected by primary oral mucosal melanoma [38, 39].

It has been suggested that in metastatic melanoma there will be proliferation of metastatic melanoma cells in the lamina propria/submucosa without any epithelial/lamina proprial junctional activity or invasion of the epithelium, while primary melanoma will always show junctional activity [38]. However, this is not true as metastatic melanoma cells can show junctional activity [6], and primary melanoma cells originating from immature melanocytes residing in the lamina propria may proliferate and invade deeply without anything resembling intraepithelial or junctional activity [14]. To further complicate matters, if in a subject with cutaneous melanoma oral mucosal melanoma was subsequently to develop, it might be assumed that this must be metastatic from the cutaneous melanoma; but it may well be an independent second primary tumour. Only genetic molecular investigation can solve this uncertainty, but the issue is academic rather than practical.

## 8. Conclusion

Owing to the rarity of oral mucosal melanoma, there is little evidence-based information about its pathogenesis and its treatment, though there is overwhelming evidence that, despite any treatment, the outcome is invariably fatal.

It is clear that susceptibility to oral mucosal melanoma is a polygenetic trait with genetic variants of MC1R gene and gain-of-function mutations of the* KIT* receptor tyrosine kinase playing important roles; and melanin itself and byproducts of melanin biosynthesis all contribute to the overall carcinogenic effect. It seems that the extrinsic risk factors widely known to be associated with carcinogenesis have little capacity to modify the process of melanomagenesis.

## Figures and Tables

**Table 1:** Differential diagnoses of mucosal melanoma [1, 6, 8].

(1) Oral mucosal melanin hyperpigmentation
(i) Physiological (racial)
(ii) Melanotic maculae
(iii) Melanoacanthoma
(iv) Melanotic nevus
(v) Tobacco-induced
(vi) Drug-induced
(vii) Inflammation related
(viii) Associated with syndromes or systemic disease (Peutz-Jegher syndrome, McCune-Albright syndrome, Laugier-Hunziker syndrome, Addison disease, neurofibromatosis)

(2) Angioproliferative disorders
(i) Haemangioma
(ii) Vascular malformations
(iii) Kaposi sarcoma

(3) Extrinsic pigment
(i) Amalgam tattoo
(ii) Recreational tattoo

(4) Benign inflammatory, reactive, neoplastic growths that should be differentiated from amelanotic melanoma
(i) Pyogenic granuloma
(ii) Fibrous hyperplasia
(iii) Peripheral giant cell granuloma

**Table 2:** American Joint Committee on Cancer (AJCC) TNM staging of mucosal melanoma [1–3, 35].

T: primary tumour		N: regional lymph node	M: distant metastasis
T3: mucosal disease		NX: regional lymph nodes that cannot be assessed	MO: no distant metastasis

T4a: moderately advanced disease involving deep soft tissues, bone, and overlying skin		NO: no regional lymph node metastasis	M1: distant metastasis present

T4b: very advanced disease involving brain, dura, skull base, lower cranial nerves (IX, X, XI, and XII), masticator space, paravertebral space, or mediastinal structures		N1: regional lymph node metastasis present	

Different clinical stages of oral mucosal melanoma			

Stage	T	N	M

Stage III	T3	NO	MO

Stage IVa	T4a	NO	MO
	or T3-T4a	N1	MO

Stage IVb	T4	Any N	MO

Stage IVc	Any T	Any N	M1

## Abbreviations

SCF: Stem cell factor
MC1R: Melanocortin receptor 1
JAK: Janus Kinase
*α*MSH: *α*-Melanocyte stimulating hormone
STAT: Signal Transduction and Activator of Transcription
TNM: Tumour/lymph node/metastases
RTK: Receptor tyrosine kinase
PI3K: Phosphoinositide 3′-kinase
POMC: Proopiomelanocortin
AJCC: American Joint Committee on Cancer.

## References

[1] Chatzistefanou I., Kolokythas A., Vahtsevanos K., Antoniades K. (2016). Primary mucosal melanoma of the oral cavity: current therapy and future directions. *Oral Surgery, Oral Medicine, Oral Pathology and Oral Radiology*.

[2] Breik O., Sim F., Wong T., Nastri A., Iseli T. A., Wiesenfeld D. (2016). Survival outcomes of mucosal melanoma in the head and neck: case series and review of current treatment guidelines. *Journal of Oral and Maxillofacial Surgery*.

[3] Bakkal F. K., Basman A., Kizil Y. (2015). Mucosal melanoma of the head and neck: recurrence characteristics and survival outcomes. *Oral Surgery, Oral Medicine, Oral Pathology and Oral Radiology*.

[4] Pfister D. G., Ang K., Brizel D. M. (2012). Mucosal Melanoma of the Head and Neck. *Journal of the National Comprehensive Cancer Network*.

[5] Kottschade L. A., Grotz T. E., Dronca R. S. (2014). Rare presentations of primary melanoma and special populations: a systematic review. *American Journal of Clinical Oncology*.

[6] Femiano F., Lanza A., Buonaiuto C., Gombos F., Spirito F. D., Cirillo N. (2008). Oral malignant melanoma: a review of the literature. *Journal of Oral Pathology and Medicine*.

[7] Mohan M., Sukhadia V. Y., Pai D., Bhat S. (2013). Oral malignant melanoma: systematic review of literature and report of two cases. *Oral Surgery, Oral Medicine, Oral Pathology and Oral Radiology*.

[8] Smith M. H., Bhattacharyya I., Cohen D. M. (2016). Melanoma of the oral cavity: an analysis of 46 new cases with emphasis on clinical and histopathologic characteristics. *Head and Neck Pathology*.

[9] Lopez F., Rodrigo J. P., Cardesa A. (2016). Update on primary head and neck mucosal melanoma. *Head and Neck*.

[10] Feller L., Masilana A., Khammissa R. A. G., Altini M., Jadwat Y., Lemmer J. (2014). Melanin: the biophysiology of oral melanocytes and physiological oral pigmentation. *Head and Face Medicine*.

[11] Feller L., Chandran R., Kramer B., Khammissa R. A. G., Altini M., Lemmer J. (2014). Melanocyte biology and function with reference to oral melanin hyperpigmentation in HIV-seropositive subjects. *AIDS Research and Human Retroviruses*.

[12] Chandran R., Khammissa R. A. G., Lemmer J., Feller L. (2014). HIV-associated oral melanin hyperpigmentation. *Journal of the South African Dental Association*.

[13] Masilana A., Khammissa R. A. G., Lemmer J., Feller L. (2015). Oral medicine case book 66: Physiological/racial oral melanin hyperpigmentation. *Journal of the South African Dental Association*.

[14] Tlholoe M. M., Khammissa R. A. G., Bouckaert M., Altini M., Lemmer J., Feller L. (2015). Oral mucosal melanoma: some pathobiological considerations and an illustrative report of a case. *Head and Neck Pathology*.

[15] Feller L., Bouckaert M., Chikte U. M. (2010). A short account of cancer—specifically in relation to squamous cell carcinoma. *Journal of the South African Dental Association*.

[16] Grichnik J. M., Rhodes A. R., Sober A. J., Sydor A. M., Pancotti R. (2016). Beign neoplasias and hyperplasias of melanocytes. *in Fitzpatrick's Dermatology in General Medicine*.

[17] Torres-Cabala C. A., Wang W.-L., Trent J. (2009). Correlation between KIT expression and KIT mutation in melanoma: a study of 173 cases with emphasis on the acral-lentiginous/mucosal type. *Modern Pathology*.

[18] White R. M., Zon L. I. (2008). Melanocytes in development, regeneration, and cancer. *Cell Stem Cell*.

[19] Papaspyrou G., Garbe C., Schadendorf D., Werner J. A., Hauschild A., Egberts F. (2011). Mucosal melanomas of the head and neck: New aspects of the clinical outcome, molecular pathology, and treatment with c-kit inhibitors. *Melanoma Research*.

[20] Heinrich M. C., Blanke C. D., Druker B. J., Corless C. L. (2002). Inhibition of KIT tyrosine kinase activity: a novel molecular approach to the treatment of KIT-positive malignancies. *Journal of Clinical Oncology*.

[21] Curtin J. A., Busam K., Pinkel D., Bastian B. C. (2006). Somatic activation of KIT in distinct subtypes of melanoma. *Journal of Clinical Oncology*.

[22] Seetharamu N., Ott P. A., Pavlick A. C. (2010). Mucosal melanomas: a case-based review of the literature. *Oncologist*.

[23] Buery R. R., Siar C. H., Katase N. (2011). NRAS and BRAF mutation frequency in primary oral mucosal melanoma. *Oncology Reports*.

[24] Rivera R. S., Nagatsuka H., Gunduz M. (2008). C-kit protein expression correlated with activating mutations in KIT gene in oral mucosal melanoma. *Virchows Archiv*.

[25] Feller L., Khammissa R. A. G., Kramer B., Altini M., Lemmer J. (2016). Basal cell carcinoma, squamous cell carcinoma and melanoma of the head and face. *Head and Face Medicine*.

[26] Bandarchi B., Jabbari C. A., Vedadi A., Navab R. (2013). Molecular biology of normal melanocytes and melanoma cells. *Journal of Clinical Pathology*.

[27] Garzino-Demo P., Fasolis M., Maggiore G. M. L. T., Pagano M., Berrone S. (2004). Oral mucosal melanoma: a series of case reports. *Journal of Cranio-Maxillofacial Surgery*.

[28] Kahn M. A., Weathers D. R., Hoffman J. G. (2005). Transformation of a benign oral pigmentation to primary oral melanoma. *Oral Surgery, Oral Medicine, Oral Pathology, Oral Radiology and Endodontology*.

[29] Shen Z.-Y., Liu W., Bao Z.-X., Zhou Z.-T., Wang L.-Z. (2011). Oral melanotic macule and primary oral malignant melanoma: epidemiology, location involved, and clinical implications. *Oral Surgery, Oral Medicine, Oral Pathology, Oral Radiology and Endodontology*.

[30] Feller L., Masipa J. N., Wood N. H., Khamissa R. A., Meyerov R., Lemmer J. (2008). Primary oral melanoma associated with HIV infection. *Journal of the South African Dental Association*.

[31] Luna-Ortiz K., Aguilar-Romero M., Villavicencio-Valencia V. (2016). Comparative study between two different staging systems (AJCC TNM VS BALLANTYNE'S) for mucosal melanomas of the Head & Neck. *Medicina Oral Patologia Oral y Cirugia Bucal*.

[32] Tomic K., Mihajlovic G., Jankovic S., Djonovic N., Jovanovic-Mihajlovic N., Diligenski V. (2012). Primary mucosal melanomas: a comprehensive review. *International Journal of Clinical and Experimental Pathology*.

[33] Kim C.-Y., Kim D. W., Kim K., Curry J., Torres-Cabala C., Patel S. (2014). GNAQ mutation in a patient with metastatic mucosal melanoma. *BMC Cancer*.

[34] Ugurel S., Hildenbrand R., Zimpfer A. (2005). Lack of clinical efficacy of imatinib in metastatic melanoma. *British Journal of Cancer*.

[35] Wang X., Wu H.-M., Ren G.-X., Tang J., Guo W. (2012). Primary oral mucosal melanoma: advocate a wait-and-see policy in the clinically no patient. *Journal of Oral and Maxillofacial Surgery*.

[36] Feller L., Lemmer J. (2011). Cancer metastasis: a short account. *Journal of the South African Dental Association*.

[37] Feller L., Lemmer J. (2012). 'second primary' cancers. *Journal of the South African Dental Association*.

[38] Leandro Santos R. S., Andrade M. F., de Abreu Alves F., Kowalski L. P., da Cruz Perez D. E. (2016). Metastases of melanoma to head and neck mucosa: a report of short series. *Clinical and Experimental Otorhinolaryngology*.

[39] Tas F., Keskin S. (2013). Mucosal melanoma in the head and neck region: different clinical features and same outcome to cutaneous melanoma. *ISRN Dermatology*.

